# A Case of Acquired Nerve Deafness

**Published:** 1902-06-07

**Authors:** W. D. Haslam


### Hospital Clinics and Medical Progress.

### A CASE OF ACQUIRED NERVE DEAFNESS.

By W. D. Haslam, M.D., M.R.C.S.

*Mrs. G., æt.* 50, Carshalton, has been married for 28 years: no children. Came under treatment on June 30th, 1899, for incontinence of urine and deafness. The following notes comprise a brief summary of the case.

The patient was a stout, plethoric woman in fairly good health, but complained of a general sense of languor, with aching across lumbar regions; she had a worried, anxious expression.

It was elicited from her that she had been deaf for nine years. The deafness came on gradually, without pain or discharge. A whistling, hissing tinnitus was the earliest symptom: the left ear was affected about a year before the right. Tinnitus, which was at first occasional, gradually became more constant; in like manner deafness gradually supervened, becoming, as time went on, worse, but its intensity varied, for some days she could hear better than others. But the main feature of her case was tinnitus and deafness coming on without assignable cause, gradually becoming worse, until deafness was almost absolute, and the tinnitus harrassed her night and day. After suffering from these distressing symptoms for three years incontinence of urine began to trouble her; first it was nocturnal, afterwards it became continuous.

*Present Condition,* July 4th, 1899.—Hearing power for ordinary conversation is lost. The watch-tick could not be heard on contact. The vibrating tuning-fork could only be heard at its loudest for a few seconds, and I noted down that Rinne's test was minus.

The pharynx was relaxed; nasal respiration was normal.

Formerly she could hear better "in the quiet" than in a noise. She could, in conversation, follow one voice, but two produced a painful sense of confusion. Through the speculum the membrana tympani was slightly retracted, had lost its lustre, and was thickened.

The patient never had special treatment, and had long since given up her case as hopeless; but she said "the noises in her head were enough to drive her out of her mind." The incontinence of urine, in the meantime, had become so serious that she was willing to submit to any treatment that would be likely to procure relief. Her condition rendered her almost a prisoner to the house, and cut her off from all social intercourse.

Thinking there might be some pressure on the bladder, I made a vaginal examination and found none. The uterus was undeveloped—infantile—probably the ovaries were absent as patient had never menstruated. There was no paralysis of the sphincter ani or of the muscular system generally.

*Diagnosis.*—The deafness appeared to be partly catarrhal and partly nervous.

I therefore ordered inhalations and applied astringents to the pharynx, but relied chiefly on bromide of ammonia with strychnine internally.

This course of treatment was continued with little or no alteration for three months; by the end of August the tinnitus was almost subdued, and I saw less of the patient in consequence.

As regards the incontinence of urine I had to rely upon strychnine.

As the patient visited me from time to time, I found the tuning-fork tests were contradictory; sometimes Rinne was minus, at others it appeared to be plus. Thinking that the nerve deafness was functional, I ordered an ear trumpet in September, and she was now enabled to enter into conversation. After the tinnitus subsided, the hearing power was distinctly improved, and the patient was left to continue the treatment (iron and strychnine).

By the end of 1900, I find from my notes the patient, who a year ago could not hear her own clock tick, could then sit in her house, with closed doors, and hear the distant church clocks strike; her hearing power for ordinary conversation was almost equal to normal, for she is able to go to church and hear every word.

*April 4th,* 1902.—I called on this patient to-day and found her in better health than she had been for years, and there was no apparent deafness.

This case, I think, is interesting on account of the chain of events which followed one another with such marked precision. First there was an attack of catarrhal deafness, gradually becoming worse and leading on to acquired nerve-deafness; then the distressing tinnitus constantly harassing the nervous system, causing an increased weakness of the auditory nerve already enfeebled by want of use.

Not only was this so, but I think I am justified in supposing that the tinnitus, constantly harassing the nervous system, also brought about a paretic condition of the bladder, whereby its power of retention was abolished.

It is also not of little interest to find how all these symptoms gradually disappeared under the use of nerve tonics, and the patient is well rewarded for persevering as she did with the remedies over a long course of treatment. The daily use of the ear trumpet appeared to be very beneficial in restoring the lost function of the auditory apparatus.

There was not a trace of hysteria in this case. The patient's general health began to improve directly the noises ceased.

